# Attitudes and Usage of ChatGPT Among Medical and Paramedical Students in Iran: A Cross‐Sectional Study

**DOI:** 10.1002/hsr2.72899

**Published:** 2026-07-30

**Authors:** Seyyedeh Fatemeh Mousavi Baigi, Masoumeh Sarbaz, Kosar Ghaddaripouri, Khalil Kimiafar

**Affiliations:** ^1^ Department Health Information Technology, School of Paramedical and Rehabilitation Sciences Mashhad University of Medical Sciences Mashhad Iran; ^2^ Student Research Committee Mashhad University of Medical Sciences Mashhad Iran; ^3^ Student Committee of Medical Education Development, Education Development Center Mashhad University of Medical Sciences Mashhad Iran; ^4^ Health Information Management Department Shiraz University of Medical Sciences Shiraz Iran; ^5^ Student Research Committee Shiraz University of Medical Sciences Shiraz Iran

**Keywords:** artificial intelligence, machine learning, education, technology, ChatGPT, large language models

## Abstract

**Background and Aims:**

ChatGPT, an AI‐powered conversational tool, has shown potential to transform healthcare education. However, its integration poses challenges regarding usability, ethics, and critical thinking. Understanding students' attitudes and usage patterns is vital for successful adoption. The aim of this study was to translate and psychometrically validate the TAME‐ChatGPT (Technology Acceptance Model Edited to Assess ChatGPT Adoption) tool into Persian and to evaluate the attitudes and usage of this technology among medical and paramedical students at Mashhad University of Medical Sciences (MUMS).

**Methods:**

This cross‐sectional study was conducted over 2 months (December 21, 2024–February 21, 2025) at MUMS. A TAM‐based questionnaire was administered to 246 students. The instrument was validated through forward‐backward translation, content validity (CVI = 0.90), exploratory factor analysis, and reliability testing (Cronbach's alpha = 0.82–0.88). Data analysis, performed using SPSS 27, included Chi‐square, Mann‐Whitney U, and regression analyses. Thematic analysis was applied to qualitative responses.

**Results:**

A total of 246 students participated (response rate: 100%). Among participants, 86.6% were aware of ChatGPT, and 65.5% reported prior usage. Awareness and usage were both significantly higher among male students (*p* = 0.002 and *p* = 0.007, respectively). Positive attitudes were observed toward ease of use (mean = 4.06, SD = 0.80) and usefulness (mean = 4.03, SD = 0.77), though concerns persisted about critical thinking (mean = 3.38, SD = 1.11) and perceived risks. Regression analysis showed academic discipline as a key predictor, with health information technology students having the highest acceptance (β = 0.28, *p* < 0.01). Key challenges included access issues, response quality, and training needs.

**Conclusions:**

Students had positive attitudes toward ChatGPT's usability and potential. Addressing ethical concerns and critical thinking is essential. Tailored training and policies are required for responsible integration in healthcare education.

## 1 Introduction

The integration of artificial intelligence (AI) and machine learning (ML) into healthcare education has revolutionized medical training, enhancing personalized learning, critical thinking, and evidence‐based decision‐making [1, 2, 3]. Among AI‐driven innovations, ChatGPT, developed by OpenAI, has emerged as a valuable tool for facilitating human‐like interactions, problem‐solving, and academic research support in medical education [4, 5, 6, 7].

Despite its potential, concerns regarding ethical implications, data security, plagiarism, bias, and misinformation remain [8, 9, 10]. Studies have demonstrated ChatGPT's utility in expanding accessibility and improving educational delivery across diverse linguistic and academic contexts [11, 12]. For instance, Jaber et al. (2024) identified key enablers and barriers influencing AI adoption among healthcare professionals, underscoring the need for regionally tailored implementation strategies [13].

The technology acceptance model (TAM) provides a structured approach to understanding AI adoption in educational settings by assessing perceived usefulness, ease of use, and behavioral intentions [14, 15, 16, 17]. Prior research suggests TAM is particularly effective in low‐ and middle‐income countries (LMICs), where socio‐economic constraints impact technology adoption [18, 19, 20]. While healthcare students often express positive attitudes toward AI, challenges such as limited knowledge and training hinder effective implementation [21, 22].

Despite increasing global research, a knowledge gap persists regarding AI adoption in LMICs, particularly Iran, where socio‐economic factors necessitate localized investigations. A systematic review by Mousavi Baigi et al., found that although most healthcare students hold a favorable view of AI in clinical contexts, their actual knowledge and skills remain insufficient. The authors emphasized the need for structured face‐to‐face instruction and clear training materials to bridge this gap [23]. Similarly, Hasan et al. explored ethical concerns regarding AI implementation among pharmacists across the MENA region. Their findings revealed deep apprehensions about data privacy, cybersecurity, potential job displacement, and insufficient legal regulation—highlighting the importance of ethical frameworks in technology adoption [24]. Sallam et al. conducted a descriptive study across medical, dental, pharmacy, and public health disciplines, illustrating both the pedagogical benefits of ChatGPT and significant risks—such as weakened critical thinking, academic dishonesty, and lack of emotional engagement in learning environments [25].

From a broader perspective, van Dis et al., proposed five research priorities for responsible AI deployment in academia, including transparency, accountability, and value‐sensitive design [26]. Additionally, Ghaddaripouri et al. demonstrated how ML algorithms have effectively improved diagnostic and predictive capacities—particularly in the case of meningitis—reinforcing the broader utility of AI in healthcare delivery and medical training [27].

Against this backdrop, the present study aims to translate and validate the Persian version of the TAME‐ChatGPT (Technology Acceptance Model Edited to Assess ChatGPT Adoption) questionnaire and to assess the attitudes and usage patterns of medical and paramedical students at Mashhad University of Medical Sciences (MUMS). Using the TAM framework, this research seeks to inform policy and curriculum design for the ethical and effective integration of conversational AI tools in health sciences education.

## 2 Methods

### 2.1 Study Design

This analytical cross‐sectional study was conducted over a 2‐month period from December 21, 2024, to February 21, 2025, at Mashhad University of Medical Sciences (MUMS). Mashhad, the second most populous city in Iran after Tehran, is one of the country's major metropolises, located in northeastern Iran. MUMS, one of the largest and most prestigious medical universities in the country, operates under the supervision of the Ministry of Health and Medical Education. In addition to its prominent role in healthcare delivery—serving an estimated population of five million—the university is recognized as a key institution for scientific and healthcare advancements, hosting advanced educational and research centers.

The present study was approved by the Ethics Committee of Mashhad University of Medical Sciences under the ethical code IR.MUMS. REC.1403.258 on October 23, 2024. Data collection began on December 21, 2024, and was completed within a 2‐month timeframe, ending on February 21, 2025. The primary objective of the study was to investigate the attitudes and usage patterns of medical and paramedical students toward ChatGPT using the TAM framework. The Consensus‐Based Checklist for Reporting Survey Studies (CROSS) was employed to ensure comprehensive and systematic reporting of the study results [28]. All ethical principles in research, according to Helinsky's statement, have been observed in this study. After explaining the research, the participatients entered the research with informed consent. All participatient's information has been used confidentially and without disclosing names.

### 2.2 Measurement Tool & Validation: TAME‐ChatGPT

The study utilized a questionnaire adapted from the original instrument developed by Sallam et al. [29], based on the TAM. The questionnaire was employed to assess users' attitudes and behaviors regarding ChatGPT. It consisted of two main sections: First Section: Consisting of 13 questions targeting participants who were familiar with ChatGPT but had not used it. Second Section: Containing 23 questions for participants who reported having experience using ChatGPT.

All items in the questionnaire were evaluated using a five‐point Likert scale, ranging from “Strongly Disagree” (score of 1) to “Strongly Agree” (score of 5). Negatively worded items were retained in their original coding direction, and higher scores on these items therefore reflected higher perceived concerns or risks. This instrument comprehensively assessed various components of the TAM framework, including perceived usefulness, perceived ease of use, social influence, perceived risks, and behavioral factors, providing a holistic evaluation of the participants' attitudes and behaviors regarding ChatGPT.

To ensure the scientific rigor of the instrument used in this study, multiple processes were employed to evaluate the validity and reliability of the TAME‐ChatGPT questionnaire. This tool, based on the TAM, included 13 questions for participants who had only heard about ChatGPT but had not used it, and 23 questions for those who had prior experience with the tool. A meticulous process of translation and cultural adaptation was conducted to ensure its applicability to the Persian‐speaking population.

The translation process followed the standard forward‐backward translation methodology. Initially, two independent translators proficient in artificial intelligence concepts and familiar with the specialized language of the instrument translated it into Persian. Subsequently, a third translator performed a back‐translation of the Persian version into English. The back‐translated version was compared with the original by an expert panel comprising nine members: four PhD students in health information management (HIM) and health information technology (HIT), two researchers with PhDs in HIM, two researchers with PhDs in medical informatics, and one researcher with a PhD in biostatistics. This panel evaluated and refined the instrument to ensure linguistic and cultural alignment.

Finally, the finalized Persian version was approved after implementing the recommended modifications, ensuring the tool's validity and reliability for the target population.

### 2.3 Content and Face Validity

The content validity of the instrument was evaluated using the content validity ratio (CVR) and content validity index (CVI). Additionally, the face validity of the instrument was assessed by examining its clarity and readability among 10 medical and paramedical students. Feedback from these students was incorporated to finalize the questionnaire.

### 2.4 Construct Validity

Exploratory factor analysis (EFA) was conducted using principal component analysis (PCA) with Oblimin rotation to evaluate the factor structure of the instrument.

### 2.5 Reliability

The internal consistency of the instrument was evaluated using Cronbach's alpha. Additionally, test–retest reliability was assessed using the intraclass correlation coefficient (ICC).

### 2.6 Sample Size

This study included two target populations: general medical students (preclinical, physiopathology, externship, and internship stages) and paramedical students (radiology, social work, occupational therapy, health information technology, laboratory sciences, optometry, physiotherapy, and speech therapy) at Mashhad University of Medical Sciences.

The sample size was calculated separately for each population using Cochran's formula for sample size estimation:

$$n=\frac{Z^{2}\times p\times \left(1-p\right)}{2_{d}}$$

where Z = 1.96 (for 95% confidence level), *p* = 0.5 (maximum variability), and d = 0.05 (margin of error).

Based on this formula, the sample size was determined as 96 for medical students and 87 for paramedical students.

Subsequently, a proportional stratified sampling method was applied to allocate the total sample size to various subgroups (disciplines and educational levels) to ensure representative coverage. The number of participants per subgroup was calculated by:

$$i^{n}=n\times \frac{i^{N}}{N}$$

where _i_n is the sample size for subgroup i, _i_N is the population size of subgroup i, N is the total population size, and n is the total sample size for each population. Overall, a total of 246 students were selected according to this method, reflecting the diversity of the target population.

Eligible students within each stratum were randomly selected using official university enrollment lists. Invitations to participate in the study were distributed via official university email accounts and student social media groups affiliated with each school of study. The study objectives and participation details were explained in the invitation message, and an electronic informed consent form was obtained prior to questionnaire completion. To enhance participation and reduce non‐response bias, students who completed the questionnaire were entered into a lottery draw for a small incentive (a flash memory drive). Reminder messages were sent up to three times at separate intervals to students who had not yet responded. If a selected student did not complete the questionnaire after three reminders, they were considered non‐respondents. Through this structured follow‐up process, all selected students completed the questionnaire, resulting in a final sample of 246 participants and a response rate of 100%.

This sample size was considered adequate for EFA given the number of questionnaire items (23), although due to sample size limitations, confirmatory factor analysis (CFA) was not conducted and is acknowledged as a limitation of the study.

### 2.7 Statistical Analysis

Statistical analyses were conducted using SPSS version 27. Descriptive statistics included mean, median, standard deviation, and the Shapiro–Wilk test to assess normality. Because several variables were not normally distributed, non‑parametric tests were applied for group comparisons. For inferential analyses, the chi‑square (χ^2^) test was used for categorical variables, and the Mann–Whitney U test was applied for comparisons involving non‑normally distributed scale variables. Multiple regression analysis was performed to examine the influence of gender, academic level, and field of study on perceived risks, positive attitudes, and perceived ease of use. Standardized beta coefficients (β) were reported to facilitate interpretation of effect size. All statistical tests were two‑sided, and a priori statistical significance was set at *p* < 0.05. Subgroup and discipline‑level analyses were considered exploratory and were not prespecified in the original study design.

Thematic analysis was applied to qualitative data obtained from open‑ended survey responses. Two independent analysts coded the responses and resolved discrepancies through discussion to enhance analytical reliability. Key themes were synthesized and reported in the results [30].

## 3 Results

### 3.1 Participant Characteristics

A total of 246 responses were collected, achieving a response rate of 100%. The mean age of the study sample was 22 years (SD = 3.4). The characteristics of the study participants are summarized in Table 1. Among the 246 participants, 213 (86.6%) reported being aware of ChatGPT prior to the study, and 161 (65.5%) indicated having used ChatGPT before participating in the study.

**Table 1:** Characteristics of study respondents (*N* = 246).

Variable	Subcategories	Frequency (*n*)	Percentage (%)
Gender	Female	167	67.9
	Male	79	32.1
Marital status	Married	32	13
	Single	214	87
Education level	PhD	5	2
	Clinical Specialty Doctorate	2	0.8
	General Doctorate	118	48
	Master's Degree	18	7.3
	Bachelor's Degree	103	41.5
School of study	Medical	122	49.6
	Paramedical	124	50.4
Awareness of ChatGPT	Yes	213	86.6
	No	33	13.4
Usage of ChatGPT	Yes	161	65.5
	No	85	34.5

### 3.2 Psychometric Properties of the Persian TAME‐ChatGPT Questionnaire

Content and face validity of the Persian version of the TAME‐ChatGPT questionnaire were assessed by an expert panel consisting of nine specialists in health information management, health information technology, medical informatics, and biostatistics. The CVR values for all items exceeded the acceptable threshold of 0.78, indicating that all items were considered essential. The CVI was also evaluated in terms of relevance, clarity, and simplicity, with an overall average CVI of 0.90. Face validity was confirmed through feedback obtained from 10 medical and paramedical students, who reported that the questionnaire items were clear and understandable.

Construct validity was evaluated using EFA with principal component analysis and Oblimin rotation. The Kaiser–Meyer–Olkin (KMO) measure of sampling adequacy was 0.823 for the attitude scale and 0.702 for the usage scale, indicating acceptable adequacy for factor analysis. Bartlett's test of sphericity was statistically significant (*p* < 0.001), confirming the suitability of the data for factor analysis. The EFA identified three dimensions for the attitude scale (perceived risks, social influence, and anxiety), explaining 69.3% of the total variance. For the usage scale, four dimensions were identified (perceived usefulness, perceived ease of use, perceived risks, and behavioral factors), accounting for 72% of the variance.

Reliability analysis demonstrated high internal consistency across the questionnaire dimensions. Cronbach's alpha coefficients ranged from 0.82 to 0.88 across the different constructs, indicating strong internal reliability. In addition, test–retest reliability analysis conducted on a subsample of 30 students over a 2‐week interval showed intraclass correlation coefficients ranging from 0.84 to 0.91, demonstrating excellent temporal stability of the instrument.

### 3.3 Awareness of ChatGPT

According to Table 2, the results revealed that awareness of ChatGPT was significantly higher among males compared to females (*p* = 0.002). Specifically, 96.2% (76/79) of men versus 82.0% (137/167) of women reported having heard about ChatGPT. Regarding the level of education, students in the Doctor of Medicine (MD) program and those in undergraduate programs demonstrated higher awareness of ChatGPT compared to students in other educational levels; however, this difference was not statistically significant (*p* = 0.249).

**Table 2:** Association between study variables and awareness of ChatGPT.

Variable	Heard about ChatGPT? Yes (*n*, %)	Heard about ChatGPT? No (*n*, %)	*χ* ^2^	*p*‐value
Gender	Women: 137/167 (82.0%) Men: 76/79 (96.2%)	Women: 30/167 (18.0%) Men: 3/79 (3.8%)	*χ* ^2^(1) = 9.27	0.002
Educational level	PhD: 5/5 (100.0%) MSc: 17/18 (94.4%) MD: 109/118 (89.8%) BSc: 82/105 (80.4%) Clinical Specialty Doctorate: 3/3 (100%)	PhD: 0/5 (0.0%) MSc: 1/18 (5.6%) MD: 12/118 (10.2%) BSc: 20/105 (19.6%) Clinical Specialty Doctorate: 0/3 (0.0%)	*χ* ^2^(4) = 6.64	0.249
School of study	Medical: 110/122 (90.2%) Paramedical: 103/124 (83.1%)	Medical: 12/122 (9.80%) Paramedical: 21/124 (16.90%)	*χ* ^2^(1) = 2.67	0.102

When students were compared by school of study, awareness was slightly higher among medical students than paramedical students, although the difference was not statistically significant (*p* = 0.102). Among medical students, the highest awareness of ChatGPT was reported by students in the basic sciences phase, whereas students in the physiopathology phase had the lowest level of awareness (*p* = 0.366).

**Figure 1:**
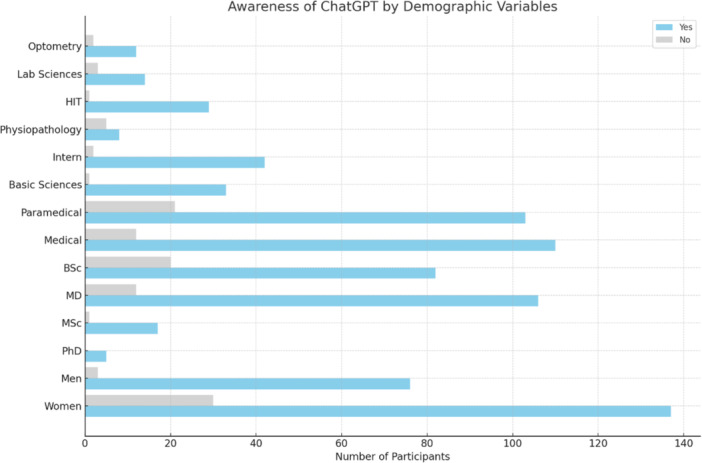
Awareness of ChatGPT by demographic variables. Percentages calculated within each subgroup (total participants, *n* = 246).

In paramedical disciplines, students majoring in HIT exhibited the highest awareness, followed by students in Laboratory Sciences. Conversely, students in other paramedical fields such as Optometry and Occupational Therapy showed lower awareness of ChatGPT (*p * = 0.094). These findings highlight variations in ChatGPT awareness based on gender, educational level, and field of study, which may be influenced by factors such as educational needs, the nature of academic training, and familiarity with technology (Figure 1).

### 3.4 Usage of ChatGPT

According to Table 3, the results showed that the use of ChatGPT was significantly higher among males compared to females (*p* = 0.007). Students in the MD and Clinical Specialty Doctorate programs, as well as those in undergraduate programs, reported relatively higher levels of ChatGPT usage compared with other educational levels; however, the difference was not statistically significant (*p* = 0.096).

**Table 3:** Association between study variables and usage of ChatGPT.

Variable	Used ChatGPT? Yes (*n*, %)	Used ChatGPT? No (*n*, %)	*χ* ^2^	*p*‐value
Gender	Women: 101/167 (60.5%) Men: 60/79 (75.9%)	Women: 66/167 (39.5%) Men: 19/79 (24.1%)	*χ* ^2^(1) = 10.03	0.007
Educational level	PhD: 3/5 (60.0%) MSc: 13/18 (72.2%) MD: 86/118 (72.9%) BSc: 57/103 (55.3%) Clinical Specialty Doctorate: 2/2 (100%)	PhD: 2/5 (40.0%) MSc: 5/18 (27.8%) MD: 32/118 (27.1%) BSc: 46/103 (44.7%) Clinical Specialty Doctorate: 0/2 (0.0%)	*χ* ^2^(4) = 16.14	0.096
School of study	Medical: 86/98 (87.8%) Paramedical: 75/96 (78.1%)	Medical: 12/98 (12.2%) Paramedical: 21/96 (21.9%)	*χ* ^2^(1) = 3.50	0.174

When students were compared by school of study, ChatGPT usage was higher among medical students (87.8%) than paramedical students (78.1%), although the difference was not statistically significant (*p* = 0.174). Among medical students, those in the basic sciences phase reported the highest use of ChatGPT, whereas students in the physiopathology phase reported the lowest usage (*p* = 0.759).

**Figure 2:**
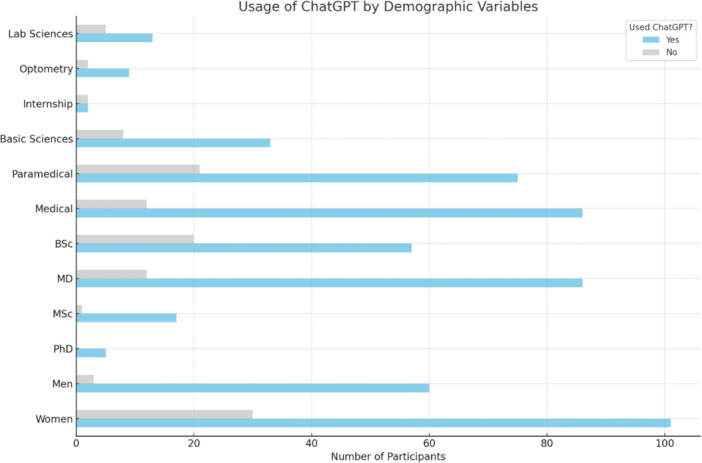
Usage of ChatGPT by demographic variables. Percentages were calculated within each school‐of‐study group among ChatGPT users (*n* = 161).

Within the paramedical disciplines, students majoring in Laboratory Sciences and HIT reported the highest levels of ChatGPT usage. Conversely, students in other paramedical fields, such as Optometry and Occupational Therapy, demonstrated lower usage of the tool (*p * = 0.164). These findings highlight differences in ChatGPT usage based on gender, educational level, and field of study, which may be influenced by factors such as the necessity of the tool for academic tasks and familiarity with emerging technologies (Figure 2).

### 3.5 Attitudes Toward ChatGPT

Attitude dimensions were calculated based on the total study sample (*n* = 246). As shown in Table 4, the means and standard deviations indicate that perceived concerns about ChatGPT included aspects such as the reliability of information (3.07 ± 1.13) and concerns about data security (3.00 ± 1.19). Among these concerns, students expressed the highest worry about the potential reduction in critical thinking skills associated with using the tool (3.38 ± 1.11). Nevertheless, positive attitudes toward the technology, including the perceived ease of use of ChatGPT (4.06 ± 0.80) and belief in its usefulness (4.03 ± 0.77), were generally high among the respondents.

**Table 4:** Means and Standard Deviations of Attitudes and Usage of ChatGPT.

Aspect	Dimension	*n*	Mean ± SD
Attitude	Concern for reliability of information	246	3.07 ± 1.13
	Concern for reduced critical thinking	246	3.38 ± 1.11
	Ease of use	246	4.06 ± 0.80
	Belief in usefulness	246	4.03 ± 0.77
Usage	Time efficiency	161	3.84 ± 0.95
	Ease of use	161	4.05 ± 0.80
	Unconscious use for assignments	161	3.11 ± 1.23
	Use as an information source	161	2.97 ± 1.25

### 3.6 Usage of ChatGPT

Usage‐related dimensions were calculated only among participants who reported prior use of ChatGPT (*n* = 161). In the analysis of means and standard deviations, students highlighted ChatGPT's practicality, particularly its ability to save time (3.84 ± 0.95) and its ease of use (4.05 ± 0.80). On the other hand, usage behaviors, such as unconscious reliance on the tool for assignments (3.11 ± 1.23) and its use as an information source (2.97 ± 1.25), were rated lower compared to other dimensions.

### 3.7 Comparison of Attitudes and Usage of ChatGPT by Gender

The analyses revealed no significant differences in perceived concerns between men and women; however, women reported slightly higher concern about the reduction in critical thinking (3.67 ± 1.15 vs. 3.43 ± 1.20, *p* = 0.15). Both genders showed similar attitudes toward the usefulness and ease of use of ChatGPT (*p* > 0.05). Regarding usage, both groups reported comparable levels of comfort in using ChatGPT (Men: 4.10 ± 0.75; Women: 4.05 ± 0.78, *p* > 0.05). Overall, no statistically significant differences were observed in attitudes or usage of ChatGPT between men and women.

### 3.8 Comparison of Attitudes and Usage of ChatGPT by School of Study and Academic Discipline

At the school‐of‐study level, no significant differences in attitudes or usage of ChatGPT were identified. For instance, the mean score for concerns about the reliability of information was similar between medical students (3.08 ± 1.12) and paramedical students (3.04 ± 1.15, *p* = 0.52).

However, significant differences were noted across various academic disciplines in terms of attitudes toward critical thinking reduction (*p* = 0.03) and the perceived importance of technology for academic success (*p* = 0.02). For instance, HIT students reported the highest mean score for the importance of technology in academic success (4.35 ± 0.85). Additionally, Laboratory Sciences students reported the highest comfort in using ChatGPT (4.20 ± 0.70, *p* = 0.04).

**Figure 3:**
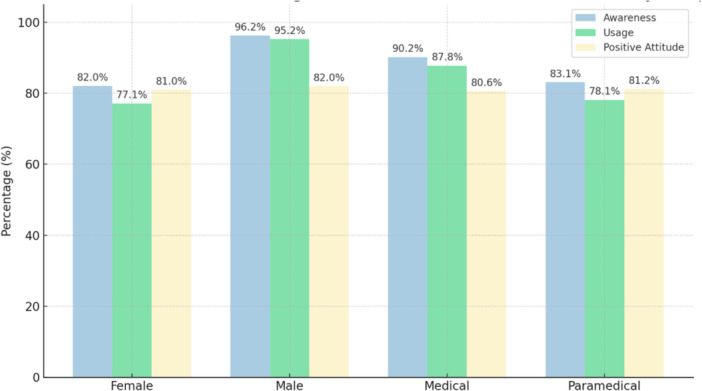
Awareness, usage, and positive attitudes toward ChatGPT by group. Percentages represent within‐group proportions of participants reporting awareness, usage, or positive attitudes toward ChatGPT (*n* = 246).

A comparative visualization of ChatGPT awareness, usage, and positive attitudes across gender and school‐of‐study groups is presented in Figure 3. This figure illustrates that while awareness and usage levels vary by group, overall positive attitudes toward ChatGPT remain consistently high among all participants.

### 3.9 Multiple Regression Analysis of Attitudes and Usage of ChatGPT

**Figure 4:**
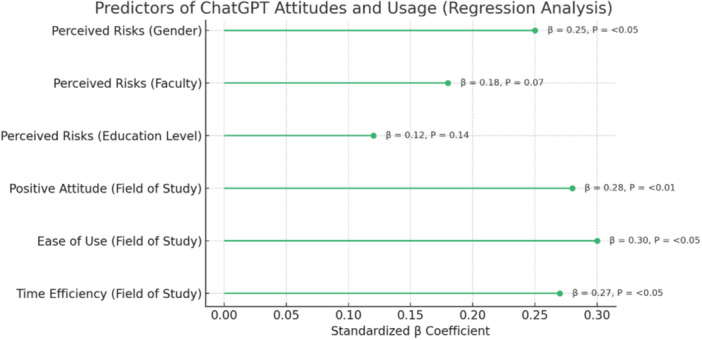
Standardized beta coefficients (β) from multiple regression models predicting perceived risks, positive attitudes, ease of use, and time‐saving capabilities. Positive β values indicate stronger positive associations between the predictor and outcome variables, while negative values indicate inverse relationships. The magnitude of β reflects the relative strength of each predictor.

This study assessed the impact of demographic variables on attitudes and usage of ChatGPT. Demographic variables included gender, school‐of‐study, academic level, and academic discipline, while dependent variables encompassed perceived risks, positive attitudes, ease of use, and time‐saving capabilities. As shown in Figure 4, the findings indicated that gender and academic discipline significantly influenced attitudes and usage of ChatGPT.

In examining perceived risks, female students demonstrated greater concern about the potential negative impacts of ChatGPT, such as reduced critical thinking (β = 0.25, *p* < 0.05). Additionally, paramedical students expressed more concerns about privacy and data security compared to medical students (β = 0.18), though this difference was not statistically significant (*p* = 0.07). Academic level did not have a significant effect on perceived risks. In this context, the coefficient for HIT students (β = 0.28) represents a moderate association according to commonly used thresholds in behavioral and educational research [31]. Therefore, the observed effects in this study (β ranging from 0.25 to 0.30) indicate practically meaningful associations.

For positive attitudes toward ChatGPT, academic discipline emerged as the strongest predictor. HIT students reported the most positive attitudes toward ChatGPT (β = 0.28, *p* < 0.01). However, gender and school‐of‐study showed no significant influence on positive attitudes.

In the analysis of ease of use, Laboratory Sciences students reported higher scores compared to other groups (β = 0.30, *p* < 0.05), while gender and school‐of‐study had no significant effect.

Finally, the analysis of time‐saving capabilities revealed that academic discipline had a significant impact. Laboratory Sciences students reported the highest scores in this domain (β = 0.27, *p* < 0.05). However, gender and school‐of‐study did not significantly influence perceptions of time‐saving benefits.

### 3.10 Qualitative Analysis of Challenges and Issues in Using ChatGPT

**Figure 5:**
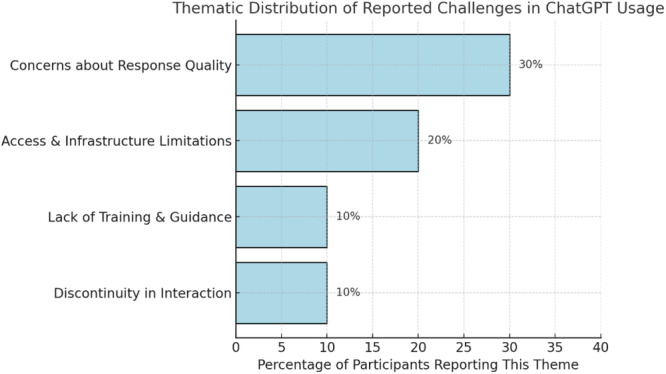
Distribution of Student‐Reported Challenges in Using ChatGPT. The bar chart depicts the percentage of participants reporting each challenge category identified through thematic analysis.

To gain deeper insight into students' experiences with ChatGPT, a thematic analysis was conducted using open‐ended survey responses. This analysis followed Braun and Clarke's six‐phase method and was reviewed independently by two coders to ensure reliability and validity. The process led to the identification of four dominant themes, which reflect practical, cognitive, and infrastructural challenges associated with ChatGPT use in educational settings (see Figure 5 for thematic distribution):

*1. Concerns about Response Quality (30%)*

The most frequently reported issue was the inconsistency and generic nature of responses. Students expressed doubts about the accuracy, depth, and relevance of ChatGPT outputs, particularly in domain‐specific medical topics. One participant stated:

Sometimes ChatGPT gives impressive‐sounding answers, but they're vague or even misleading.

*2. Access and Infrastructure Limitations (20%)*

Students emphasized barriers to access, including the need for VPNs, daily usage limitations, and high subscription costs for premium features. These constraints were particularly challenging for students from low‐resource backgrounds. Some students noted:

Accessing ChatGPT requires a VPN and often gets blocked. It's frustrating during urgent assignments.

*3. Lack of Training and Guidance (10%)*

Many respondents felt underprepared to use ChatGPT effectively, citing a lack of formal training or academic orientation on AI tools. Some recommended integrating short courses or workshops into the curriculum:

We need practical workshops to teach how to use AI tools like ChatGPT ethically and efficiently.

*4. Discontinuity in Interaction (10%)*

A subset of students reported that ChatGPT fails to maintain conversational coherence across multiple queries, which affects its usability for extended learning tasks:

It doesn't remember the context between my questions, which breaks the learning flow.

## 4 Discussion

This study represents the first effort to translate, culturally adapt, and validate the TAME‐ChatGPT instrument into Persian, providing a robust framework for assessing healthcare students' attitudes and behaviors toward ChatGPT and similar generative AI tools. The instrument demonstrated strong psychometric performance, including excellent content validity (mean CVI = 0.90; CVR > 0.78) and high internal consistency (Cronbach's α ranging 0.83–0.88), confirming conceptual clarity and cross‐cultural reliability. This methodological contribution constitutes the most significant outcome of the study, enabling standardized attitude assessment within LMICs.

Beyond methodology, several key behavioral findings emerged. First, a remarkably high proportion of students (86.6%) were aware of ChatGPT, with greater familiarity among medical students and those in technology‐driven fields such as HIT. These differences highlight how disciplinary exposure and digital literacy shape readiness to adopt AI‐based learning tools. Cross‐country comparisons mirror this pattern: studies in high‐income contexts such as Norway and the United States report similar disciplinary gradients [32, 33], whereas those in LMICs like Jordan and Pakistan associate lower awareness with infrastructural and curricular gaps [34, 35]. In Iran, the need for VPN access and bandwidth limitations imposes additional barriers, emphasizing that structural constraints can moderate technology adoption, even when attitudes are positive.

The overall attitude toward ChatGPT was favorable. Students rated both perceived usefulness (mean = 4.03 ± 0.77) and ease of use (mean  =  4.06 ± 0.80) highly, echoing global literature on AI‐assisted learning [29, 36, 37, 38]. This optimistic perception indicates growing acknowledgment of ChatGPT as a supportive educational partner—particularly for independent learning, problem‐solving, and communication training. Nevertheless, apprehensions about critical‐thinking erosion (mean  =  3.38 ± 1.11) and data security (mean  =  3.00 ± 1.19) persist. As reported elsewhere [39, 40], students in LMICs often experience elevated data‐privacy concerns owing to limited governance frameworks and unfamiliarity with institutional AI policies.

Importantly, while many participants actively used ChatGPT, the present cross‐sectional design precluded direct measurement of learning improvements. Thus, causal inference between usage frequency and educational outcomes remains speculative. This null evidence does not imply ineffectiveness: global experimental research indicates that appropriately scaffolded generative‐AI interactions can enhance motivation, self‐regulated learning, and deeper content engagement [41, 42, 43, 44]. Determining whether such benefits extend to measurable academic or clinical performance will require *longitudinal, outcome‐based studies*. Addressing this gap represents a promising future priority.

Gender‐ and discipline‐based variations also enrich the interpretation. Higher usage among male and medical students contrasts with patterns reported in Malaysia and the UAE, where female participants showed higher engagement [36, 40]. These cross‐contextual discrepancies likely reflect curricular design, cultural expectations, and access asymmetries, underscoring the need for local AI‐literacy initiatives integrated into medical curricula.

From an educational strategy standpoint, the validated Persian TAME‐ChatGPT tool can guide curricular reforms and policy decisions by furnishing a quantitative baseline for AI acceptance and ethical sensitivity within healthcare education. Developers and academic planners can use these data to tailor interventions that balance enthusiasm for innovation with vigilance about ethics, privacy, and cognitive development. Recent design studies of AI‐education applications [45] demonstrate that integrating personalized, interactive, and ethically grounded modules substantially improves learners' motivation and comprehension. Incorporating such structured digital tools—guided by instruments like TAME‐ChatGPT—could transform AI education from sporadic exposure to systematic capacity building.

Recent systematic evidence further supports the growing integration of ChatGPT in educational and healthcare environments. A recent systematic review reported generally positive attitudes toward ChatGPT among educators and healthcare professionals, highlighting its perceived usefulness for educational support, content generation, and clinical information assistance [46]. However, the review also emphasized several persistent challenges, including knowledge gaps, ethical considerations, and concerns regarding the reliability of AI generated outputs. These findings align with the present study, where students demonstrated overall positive perceptions alongside moderate concerns related to critical thinking and data security. Together, these results suggest that while enthusiasm for generative AI tools is increasing, responsible implementation will require structured training, ethical guidance, and institutional governance frameworks.

### 4.1 Strengths and Limitations

The present investigation's major strength lies in its rigorous translation–validation pipeline and its multidimensional analysis combining quantitative and qualitative evidence. High reliability coefficients, satisfactory factor structure, and thematic triangulation underscore methodological robustness and contextual relevance.

Nonetheless, several limitations warrant acknowledgment. The single‐center, convenience‐sample design restricts external generalizability, and the moderate sample size (*n* = 246) limited advanced psychometric analyses such as confirmatory factor analysis (CFA). Potential selection bias toward highly motivated or digitally active students cannot be excluded. Moreover, applying the TAM as the sole framework might omit cultural or ethical elements essential to AI adoption [19, 20, 21]. Future studies could merge TAM with approaches such as value‐sensitive design [43] to capture sociocognitive determinants of AI acceptance more holistically.

### 4.2 Future Directions

Future research should rigorously assess whether sustained ChatGPT use translates into improved learning outcomes, including analytic reasoning, academic writing, and clinical‐decision competencies. Longitudinal or quasi‐experimental designs with objective performance metrics are needed to establish causal impact.

Parallel to outcome research, capacity‐building initiatives should focus on digital‐ethics training and equitable access. Evidence from recent mobile‐app–based AI‐education projects [45] reveals that context‐specific, learner‐centered platforms combining interactivity, offline functionality, and transparent privacy policies can mitigate the digital divide. Establishing such structured, multilingual educational ecosystems could ensure ethical adoption and maximize learning benefits across resource‐diverse environments.

## 5 Conclusion

In summary, Iranian healthcare students display strong awareness and positive attitudes toward ChatGPT, tempered by ongoing ethical and infrastructural challenges. The Persian TAME‐ChatGPT instrument offers a validated foundation for longitudinal monitoring of these attitudes. While high utilization alone does not yet equate to demonstrable learning gains, purposeful, pedagogically framed integration of ChatGPT—supported by curricular design and technological facilitation—may unlock substantial educational value. Continued cross‐cultural collaboration and design‐based research will be vital to realizing this promise.

## Author Contributions

**Seyyedeh Fatemeh Mousavi Baigi:** conceptualization, investigation, methodology, visualization, writing – review and editing, writing – original draft, resources, supervision. **Masoumeh Sarbaz:** validation, methodology, writing – review and editing, resources. **Kosar Ghaddaripouri:** writing – review and editing, data curation. **Khalil Kimiafar:** conceptualization, formal analysis, writing – review and editing, supervision.

## Funding

The authors have nothing to report.

## Ethics Statement

This study was approved by the ethics committee of Mashhad University of Medical Sciences (authorization number: IR.MUMS.REC.1403.258). All ethical principles in the research were observed according to the Hellenic Declaration. All participants entered the study after receiving a full explanation of the research and providing informed consent. All information from individuals was used confidentially and without revealing their names.

## Consent

Consent for publication was obtained from all participants involved in this study. All data, including any individual information, images, or videos, were published in an anonymized form, with no personal identifying details. The data were collected and used solely for research purposes, and explicit consent was secured from the participants.

## Conflicts of Interest

The authors declare no conflicts of interest.

## 1 Transparency Statement

Seyyedeh Fatemeh Mousavi Baigi affirms that this manuscript is an honest, accurate, and transparent account of the study being reported; that no important aspects of the study have been omitted; and that any discrepancies from the study as planned (and, if relevant, registered) have been explained.

## Data Availability

The data that support the findings of this study are available from the corresponding author upon reasonable request.
